# Age and socioeconomic disparities in cervical cancer incidence and mortality: a SEER-based analysis

**DOI:** 10.3389/fpubh.2025.1591883

**Published:** 2025-09-19

**Authors:** Yuyi Ou, Santosh Chokkakula, Sio Mui Chong, Hao Wang, Andrew In-Cheong Si, Yong Jiang, Liying Huang, Xiaohua Xu, Chengliang Yin, Jun Lyu, Xiaobin Huang, Hui-ling Shang

**Affiliations:** ^1^Department of Gynaecology, Foshan Women and Children Hospital Affiliated to Guangdong Medical University, Foshan, China; ^2^Chungbuk National University College of Medicine and Medical Research Institute, Cheongju, Republic of Korea; ^3^Department of Dermatology, The University of Hong Kong-Shenzhen Hospital, Shenzhen, China; ^4^Department of Orthopedics, Clinica de Wong’s, Macao, Macao SAR, China; ^5^Department of Oncology, The University of Hong Kong-Shenzhen Hospital, Shenzhen, China; ^6^Department of Clinical Research, First Affiliated Hospital of Jinan University, Guangzhou, China; ^7^Department of Medicine, Shenzhen University, Shenzhen, China; ^8^Faculty of Medicine, Macao University of Science and Technology, Macao, Macao SAR, China; ^9^Department of Hospital Office, Foshan Women and Children Hospital Affiliated to Guangdong Medical University, Foshan, China

**Keywords:** cervical cancer, age-related patterns, incidence, mortality, SEER database

## Abstract

**Background:**

Cervical cancer (CC) remains a significant global health challenge, with marked variations in incidence and mortality influenced by age, race, and economic status. This study examines age-related patterns in CC outcomes, focusing on racial disparities and socioeconomic factors using data from the SEER18 database.

**Methods:**

We conducted a retrospective cohort study using data from the SEER 18 registries program from 2010 to 2015. Logistic regression models were used to assess factors associated with CC presence at diagnosis. Cox proportional hazard models and competing risk models examined all-cause mortality (ACM) and cancer-specific mortality (CSM). Restricted cubic spline (RCS) analysis was employed to investigate nonlinear relationships between age and CC outcomes.

**Results:**

A total of 11,183 cases of invasive cervical cancer were identified. The study revealed significant disparities in CC outcomes based on race and socioeconomic status. Black women exhibited higher incidence and mortality rates compared to White women, with this disparity widening with age. The hazard model showed that Black race (adjusted sHR 1.199, 95% CI 1.086–1.323, *p* = 0.0003) and lower income (adjusted sHR 0.842 for income over $75,000, 95% CI 0.772–0.919, *p* < 0.0001) were associated with poorer outcomes. Marital status, histological type, cancer stage, and tumor grade were also significant predictors of CC outcomes. Advanced stage (regional: adjusted sHR 3.971, 95% CI 3.517–4.483; distant: adjusted sHR 10.635, 95% CI 9.207–12.285, both *p* < 0.0001) and higher tumor grade (poorly differentiated: adjusted sHR 1.667, 95% CI 1.432–1.941; undifferentiated: adjusted sHR 1.749, 95% CI 1.363–2.244, both *p* < 0.0001) were strongly associated with increased mortality risk.

**Conclusion:**

This analysis highlights substantial racial and socioeconomic disparities in cervical cancer outcomes, exacerbated with increasing age and advanced tumor characteristics. These findings emphasize the necessity for age and population specific screening and intervention strategies to improve survival and reduce inequities among high-risk groups.

## Introduction

Cervical cancer (CC) remains a significant global health challenge, ranking fourth most common cancer among women worldwide (1). In 2020, approximately 604,127 new cases and 341,831 deaths were reported globally, with a disproportionate burden falling on lower-resource countries (2, 3). The primary cause of cervical cancer is persistent infection with high-risk types of human papillomavirus (HPV), a discovery that has revolutionized prevention strategies (4). The development of prophylactic HPV vaccines and effective cytological and HPV-based screening programs has led to substantial declines in cervical cancer incidence and mortality in many high-income countries, including the United States (5, 6).

Despite these remarkable advances, the benefits of prevention and early detection have not been distributed equitably across all populations (7). Significant disparities in cancer outcomes persist, driven by a complex web of social, economic, and structural factors (8, 9). Socioeconomic status (SES) has emerged as a powerful predictor of health outcomes for numerous cancers, including cervical cancer (10). Women with lower SES, often measured by income, education, or area-level deprivation, face numerous barriers to care, such as lack of health insurance, transportation difficulties, lower health literacy, and residence in medically underserved areas (1, 11). These factors reduce HPV vaccination uptake, hinder participation in screening programs, delay diagnosis, and impact treatment adherence, ultimately contributing to higher incidence and mortality in disadvantaged groups (12, 13).

Age is another key factor influencing CC trends. While CC incidence has historically peaked in middle age, recent reports have suggested emerging shifts in age-specific patterns. Some studies indicate that incidence rates for certain cancers are increasing in younger generations (14), and for cervical cancer, there is emerging evidence that long-term declines may be stalling or even reversing among specific subgroups of women, particularly those in low-income settings (15). Understanding these age-specific trends is crucial for tailoring public health messaging and intervention strategies to the populations at highest risk.

The intersection of SES, age, and race/ethnicity further complicates the landscape of cervical cancer disparities. Racial and ethnic minority groups, particularly Non-Hispanic Black and Hispanic women, consistently experience higher incidence and mortality from cervical cancer compared to Non-Hispanic White women (10, 16). These disparities are not rooted in biology but are manifestations of structural racism, residential segregation, and systemic inequities that result in differential access to high-quality healthcare and preventive services (17, 18).

To comprehensively characterize these intersecting disparities and inform equitable cancer control strategies, population-based analyses incorporating detailed socioeconomic and demographic data are necessary. The SEER program offers a valuable resource to examine such patterns in the United States (19). By linking cancer registry data with area-based socioeconomic indicators, it is possible to investigate disparities on a large scale. Therefore, this study leverages the SEER database to conduct a detailed analysis of the intersecting impacts of age and county-level SES on cervical cancer incidence and mortality trends in the United States from 2010 to 2015. We hypothesize that lower SES is associated with higher cervical cancer incidence and mortality, and that these disparities are most pronounced among younger women and racial/ethnic minorities, contributing to later stage at diagnosis and poorer survival.

## Methods

### Search strategy and data collection

Patient data was obtained from the publicly accessible SEER database, which includes 18 cancer registries and is available at www.seer.cancer.gov (20). We utilized SEER*Stat version 8.3.6 software for data retrieval and analysis (21). To comply with ethical and legal standards, there is a directive to expand access to the SEER Plus database. Within this framework, we focused on analyzing data from the openly accessible SEER database, which covers about 28% of the U.S. population. From this extensive resource, we extracted relevant information on patients with CC (22). The primary objective of this study is to elucidate cancer-associated determinants in CC patients diagnosed between 2010 and 2015, as per the American Joint Commission on Cancer (AJCC) Sixth Edition staging system. Since CC is reportable in all U.S. states, informed patient consent is not required. Upon signing the data usage agreement, cancer research data becomes publicly available. This work adheres to the STROCSS criteria.

### Data collection

**Figure 1:**
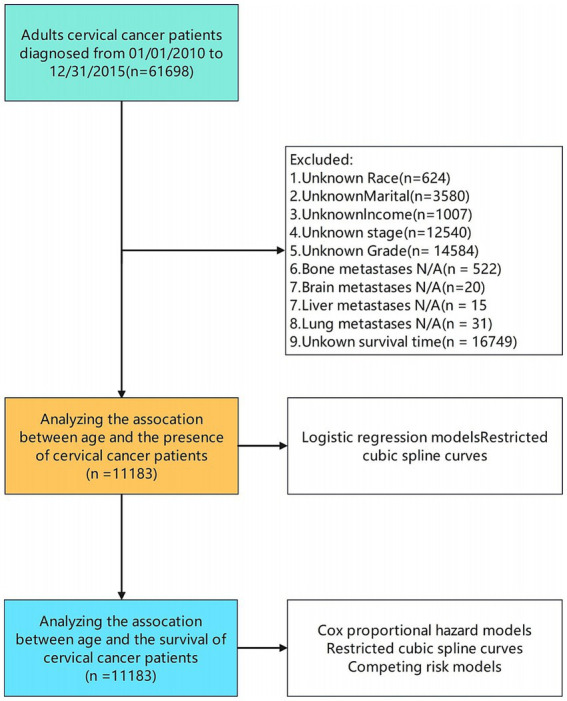
Flow diagram of patient selection process.

We identified 61,698 patients diagnosed with cervical cancer between 2010 and 2015 from the SEER database. After excluding cases with incomplete data on key variables such as race, marital status, income, AJCC staging, histologic grade, metastasis status, and survival outcomes, 11,183 patients met our inclusion criteria (Figure 1). Data extracted included demographic factors (age, gender, race, marital status, income), clinical variables [American Joint Committee on Cancer (AJCC) Sixth Edition staging, surgical treatment, radiation therapy, chemotherapy, tumor size], and vital status.

Cancer stage at diagnosis was classified according to the SEER Summary Stage system, which broadly corresponds to categories in the American Joint Committee on Cancer (AJCC) staging system. The following definitions were used. Localized stage: cancer confined to the cervix uteri without extension beyond the cervix (generally corresponds to AJCC stage I). Regional stage: cancer that has extended beyond the cervix to adjacent tissue such as the vagina or parametria and to regional lymph nodes, roughly corresponds to AJCC stages II and III. Distant stage: cancer that has spread to distant organs or non-regional lymph nodes, including metastases to the lung, liver, bone, or brain, corresponds to AJCC stage IV.

In our dataset, “lymph node involvement” refers to the recorded presence of metastasis in either regional lymph nodes or distant lymph nodes. Regional nodal metastasis denotes involvement confined to regional lymphatic stations, consistent with AJCC staging definitions. The variable labeled Metastasis corresponds exclusively to distant organ metastases, such as those affecting the liver, lung, brain, or bone. These were coded using SEER’s “CS Mets at DX” and site-specific metastasis indicators. This distinction allows differentiation between locoregional progression, which includes local and regional nodal spread, and systemic dissemination involving distant lymph nodes or organs.

### Logistic regression and cox proportional hazard models

Our logistic regression models utilized the presence of CC at diagnosis as the primary endpoint. For the Cox proportional hazard models, we examined all-cause mortality (ACM) and cancer-specific mortality (CSM), employing ICD-10 codes to determine the cause of death. Competing risk models, analyzed using proportional subdistribution hazards models, focused on cancer-specific mortality as the primary endpoint, with other mortality causes acting as competing risks (23, 24). The time to event was calculated in months, from the date of diagnosis until the end of follow-up or death.

### Restricted cubic spline analysis

We utilized restricted cubic spline (RCS) analysis as our primary method to investigate nonlinear relationships, a technique widely recognized in the field (25, 26). Previous studies recommend using 3–5 knots to balance flexibility and over-fitting, with four knots being a common choice for capturing complex nonlinear patterns while maintaining model stability (27). For population characteristics, we employed the chi-square test for categorical variables, while continuous variables were expressed as means with standard deviations and compared using *t*-tests. These analytical approaches formed the basis for our results interpretation and evaluation.

We evaluated associations between demographic and clinical factors and cervical cancer (CC) presence at diagnosis using logistic regression with restricted cubic spline (RCS) curves to model nonlinear effects of age. RCS curves with knots at the 5th, 45th, 65th, and 90th age percentiles allowed assessment of age as a continuous variable (28). Variables showing significant univariable associations (*p* ≤ 0.05) were included in multivariable models, followed by sensitivity analyses. Using the age threshold identified by RCS, patients were stratified into two groups for comparison using *t*-tests and chi-square tests. Because age showed linear associations on both sides of the threshold, multivariable logistic regression estimated adjusted risk ratios with 95% confidence intervals.

### The survival analysis

We employed univariable and multivariable Cox proportional hazards regression models to calculate mortality hazard ratios, adjusting for potential confounders (22). Restricted cubic spline models were fitted to Cox models using 4 knots at the 5th, 45th, 65th, and 90th age percentiles (28). We further adjusted ASM and CSM spline models for variables showing significance in respective univariable Cox regressions. To better estimate cervical cancer-specific mortality (CSM) and account for high competing event rates, we conducted competing mortality risk regression analysis using Fine and Gray models (29, 30). We calculated unadjusted and adjusted subdistribution hazard ratios (sHR) with 95% CI. The cumulative incidence function (CIF) was used to estimate CSM incidence while considering competing risks.

### Statistical analysis

For our data analysis, we utilized the R programming language to perform various statistical tests and computations. We considered results statistically significant when two-tailed alpha values met predetermined thresholds. This approach allowed us to rigorously examine our data and draw meaningful conclusions from our findings. The statistical analyses were performed using R software (v4.4.1; http://www.R-project.org), Zstats v1.0,^1^ and Free Statistics software (v1.3) for data processing and analysis.

## Results

### Baseline characteristics of study population

We analyzed data from 11,183 cervical cancer (CC) patients diagnosed between 2010 and 2015, stratified into two age groups: ≤49 years (mean 39.0) and ≥50 years (mean 62.7) (*p* < 0.001). The demographic analysis revealed that the majority of patients (76.21%) were white, with a slightly higher proportion in Group A (78.3%) compared to Group B (74.0%) (*p* < 0.001). Regarding marital status, 44.27% of the total patients were married, with a higher percentage in Group A (47.4%) than in Group B (40.9%) (*p* < 0.001) (Table 1). In terms of cancer characteristics, Grade II tumors were most prevalent, accounting for 44.42% of all cases, with similar distributions in both age groups (*p* < 0.001). The stage distribution showed a notable difference between the groups. Localized stage cancer was predominant overall (45.51%), but it was significantly more common in Group A (56.2%) compared to Group B (34.3%) (*p* < 0.001). Analysis indicated that the majority of cases (84.7%) involved tumors larger than 4 cm. This trend was more pronounced in Group A (88.3%) compared to Group B (80.9%) (*p* < 0.001) (Table 1). These results demonstrate distinct demographic and clinical cancer profiles by age group, with potential implications for tailored management strategies.

**Table 1:** Baseline characteristics of study population.

Variables	Categories	Patients with cancer (any stage): *N* (%)	Group A* *N* (%)	Group B* *N* (%)	*p*
Age	Mean ± SD	50.52 ± 14.52	39.0 ± 6.7	62.7 ± 9.9	<0.001
Year					
	2010	1,856 (16.60%)	946 (16.5%)	910 (16.7%)	0.69
	2011	1,817 (16.25%)	950 (16.6%)	867 (15.9%)	
	2012	1,858 (16.61%)	964 (16.8%)	894 (16.4%)	
	2013	1,833 (16.39%)	910 (15.9%)	923 (16.9%)	
	2014	1,888 (16.88%)	967 (16.9%)	921 (16.9%)	
	2015	1,931 (17.27%)	994 (17.3%)	937 (17.2%)	
Race					
	White	8,523 (76.21%)	4,486 (78.3%)	4,037 (74%)	<0.001
	Black	1,428 (12.77%)	677 (11.8%)	751 (13.8%)	
	American Indian/Alaska Native	116 (1.04%)	73 (1.3%)	43 (0.8%)	
	Asian or Pacific Islander	1,116 (9.98%)	495 (8.6%)	621 (11.4%)	
Marital					
	Married	4,951 (44.27%)	2,719 (47.4%)	2,232 (40.9%)	<0.001
	Others	6,232 (55.73%)	3,012 (52.6%)	3,220 (59.1%)	
Income					
	Below $75,000+	8,082 (72.27%)	4,211 (73.5%)	3,871 (71%)	0.004
	Over $75,000+	3,101 (27.73%)	1,520 (26.5%)	1,581 (29%)	
Rural-urban distributional					
	Urban	9,889 (88.43%)	5,082 (88.7%)	4,807 (88.2%)	0.42
	Rural	1,294 (11.57%)	649 (11.3%)	645 (11.8%)	
Primary					
	Cervix uteri	8,758 (78.32%)	4,477 (78.1%)	4,281 (78.5%)	0.813
	Endocervix	2,024 (18.10%)	1,053 (18.4%)	971 (17.8%)	
	Exocervix	203 (1.82%)	104 (1.8%)	99 (1.8%)	
	Overlapping lesion of cervix uteri	198 (1.77%)	97 (1.7%)	101 (1.9%)	
Histology					
	Squamous carcinoma	8,046 (71.95%)	3,986 (69.6%)	4,060 (74.5%)	<0.001
	Adenocarcinoma	2,665 (23.83%)	1,461 (25.5%)	1,204 (22.1%)	
	Others	472 (4.22%)	284 (5%)	188 (3.4%)	
Grade					
	Well differentiated; Grade I	1,596 (14.27%)	1,014 (17.7%)	582 (10.7%)	<0.001
	Moderately differentiated; Grade II	4,967 (44.42%)	2,554 (44.6%)	2,413 (44.3%)	
	Poorly differentiated; Grade III	4,383 (39.19%)	2,073 (36.2%)	2,310 (42.4%)	
	Undifferentiated; anaplastic; Grade IV	237 (2.12%)	90 (1.6%)	147 (2.7%)	
Stage					
	Localized	5,089 (45.51%)	3,221 (56.2%)	1,868 (34.3%)	<0.001
	Regional	4,525 (40.46%)	2,004 (35%)	2,521 (46.2%)	
	Distant	1,569 (14.03%)	506 (8.8%)	1,063 (19.5%)	
Tumor size					
	≤4 cm	1,711 (15.30%)	670 (11.7%)	1,041 (19.1%)	<0.001
	>4 cm	9,472 (84.70%)	5,061 (88.3%)	4,411 (80.9%)	
Lymph node					
	No	6,352 (56.80%)	2,827 (49.3%)	3,525 (64.7%)	<0.001
	Yes	4,831 (43.20%)	2,904 (50.7%)	1,927 (35.3%)	
Metastasis					
	No	10,564 (94.46%)	5,461 (95.3%)	5,103 (93.6%)	<0.001
	Yes	619 (5.54%)	270 (4.7%)	349 (6.4%)	
Regional nodes examined					
	No	6,338 (56.68%)	2,790 (48.7%)	3,548 (65.1%)	<0.001
	Yes	4,845 (43.32%)	2,941 (51.3%)	1,904 (34.9%)	
Regional nodes positive					
	No	3,679 (32.90%)	2,284 (39.9%)	1,395 (25.6%)	<0.001
	Yes	7,504 (67.10%)	3,447 (60.1%)	4,057 (74.4%)	
Bone					
	No	10,978 (98.17%)	5,658 (98.7%)	5,320 (97.6%)	<0.001
	Yes	205 (1.83%)	73 (1.3%)	132 (2.4%)	
Brain					
	No	11,144 (99.65%)	5,718 (99.8%)	5,426 (99.5%)	0.037
	Yes	39 (0.35%)	13 (0.2%)	26 (0.5%)	
Liver					
	No	11,028 (98.61%)	5,684 (99.2%)	5,344 (98%)	<0.001
	Yes	155 (1.39%)	47 (0.8%)	108 (2%)	
Lung					
	No	10,773 (96.33%)	5,620 (98.1%)	5,153 (94.5%)	<0.001
	Yes	410 (3.67%)	111 (1.9%)	299 (5.5%)	

Group A*, age ≤49; Group B*, age ≥50.

### Age-related diagnostic patterns in cervical cancer

**Figure 2:**
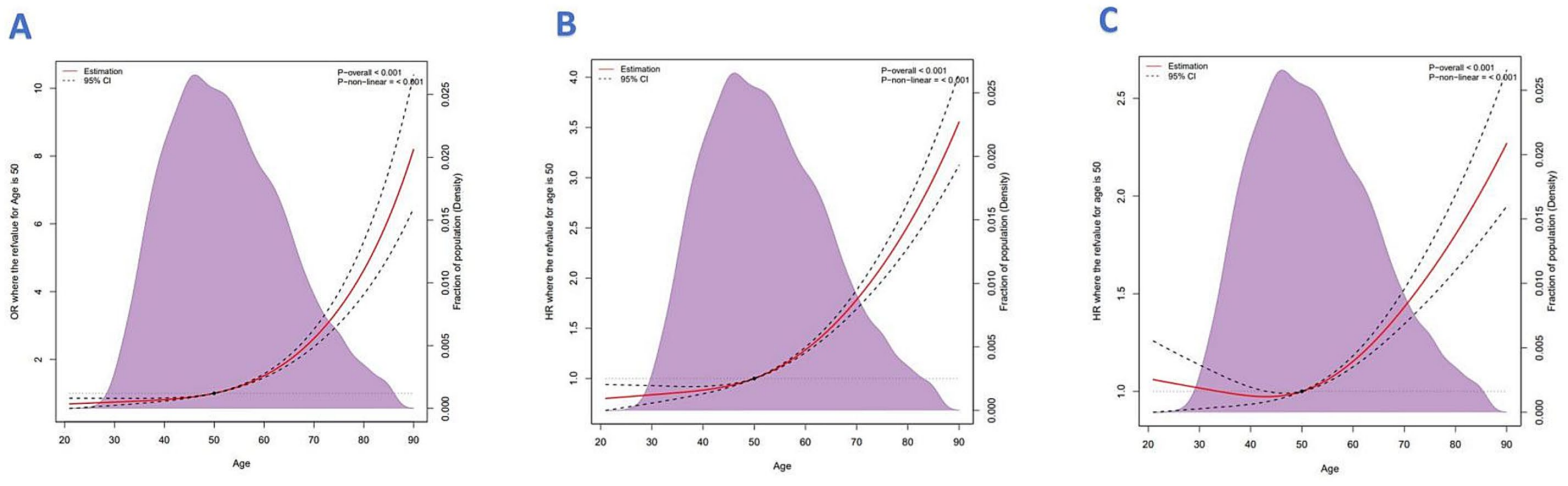
**(A)** Restricted cubic spline analysis of age vs. CC diagnosis. **(B)** All-cause mortality. **(C)** Cancer-specific mortality.

The analytic framework is shown in Figure 1. Using RCS analysis adjusted for key demographic and clinical covariates; we observed a U-shaped relationship between age and CC diagnosis (*p* < 0.001) (Figure 2A). This pattern suggests higher CC risk at both younger and older age extremes in the 2010–2015 cohort.

Univariable logistic regression identified tumor grade as a strong predictor of CC diagnosis. Compared to well-differentiated tumors, the odds ratios (OR) for moderately, poorly, and undifferentiated tumors were OR 2.30 (95% CI, 2.00–2.65), OR 4.00 (95% CI, 3.47–4.60), and OR 5.31 (95% CI, 3.99–7.07), respectively (all *p* < 0.001). These associations remained significant in multivariable models, although slightly attenuated.

Disease stage was also a key determinant. Regional-stage tumors were associated with significantly elevated odds of CC compared to localized stage (OR 5.30, 95% CI: 4.81–5.85), with distant-stage tumors exhibiting the strongest association (OR 26.43, 95% CI: 22.75–30.71, both *p* < 0.001). These relationships persisted in multivariable analysis, where distant-stage tumors had an adjusted OR of 10.00 (95% CI, 8.23–12.16, *p* < 0.001).

Interestingly, tumor size >4 cm was inversely associated with CC presence in univariable analysis (OR 0.33, 95% CI: 0.29–0.36, *p* < 0.001). The association remained statistically significant, though less pronounced, in multivariable models (OR 0.86, 95% CI: 0.75–0.97, *p* = 0.019). Lymph node involvement, typically a negative prognostic factor, showed an inverse relationship in univariable analysis (OR 0.31, 95% CI: 0.28–0.33, *p* < 0.001), but this association was not statistically significant in multivariable models (OR 1.08, 95% CI: 0.83–1.40, *p* = 0.564). Similarly, regional nodal metastasis was significant in univariable analysis (OR 3.04, 95% CI: 2.57–3.59, *p* < 0.001) but lost significance in adjusted models. These results emphasize the multifactorial nature of CC presentation and support the need for risk models that integrate histologic, staging, and demographic variables. Table 2 presents the full results of the logistic regression models.

**Table 2:** Univariable and multivariable logistic regression models for the presence of cervical cancer.

Variables		Univariable		Multivariable		Multivariable		Multivariable	
				Total		Group A*		Group B*	
		OR (95% CI)	*p*-value	OR (95% CI)	*p*-value	OR (95% CI)	*p*-value	OR (95% CI)	*p*-value
Age	Continuous	1.05 (1.05–1.05)	<0.001	1.04 (1.03–1.04)	<0.001	1.02 (1.01–1.03)	<0.001	1.05 (1.05–1.06)	<0.001
Year									
	2010	Ref.		Ref.		Ref.		Ref.	
	2011	0.90 (0.79–1.03)	0.116	0.84 (0.72–0.99)	0.033	0.92 (0.72–1.17)	0.49	0.76 (0.61–0.95)	0.016
	2012	0.92 (0.80–1.05)	0.193	0.83 (0.70–0.97)	0.018	0.95 (0.75–1.21)	0.694	0.72 (0.58–0.89)	0.003
	2013	0.88 (0.78–1.01)	0.069	0.75 (0.63–0.88)	<0.001	0.77 (0.60–0.98)	0.033	0.72 (0.58–0.89)	0.003
	2014	0.76 (0.66–0.87)	<0.001	0.62 (0.53–0.73)	<0.001	0.67 (0.52–0.85)	0.001	0.58 (0.47–0.72)	<0.001
	2015	0.71 (0.62–0.81)	<0.001	0.56 (0.48–0.66)	<0.001	0.57 (0.44–0.73)	<0.001	0.53 (0.43–0.66)	<0.001
Race									
	White	Ref.		Ref.		Ref.		Ref.	
	Black	1.77 (1.58–1.98)	<0.001	1.44 (1.26–1.66)	<0.001	1.45 (1.18–1.78)	<0.001	1.44 (1.19–1.74)	<0.001
	American Indian/Alaska Native	0.99 (0.67–1.45)	0.959	0.96 (0.62–1.50)	0.873	0.83 (0.45–1.54)	0.558	1.21 (0.62–2.36)	0.567
	Asian or Pacific Islander	0.89 (0.78–1.02)	0.099	0.86 (0.73–1.02)	0.076	1.15 (0.89–1.50)	0.288	0.75 (0.61–0.93)	0.007
Marital									
	Married	Ref.		Ref.		Ref.		Ref.	
	Others	1.63 (1.50–1.76)	<0.001	1.32 (1.19–1.45)	<0.001	1.34 (1.16–1.55)	<0.001	1.21 (1.06–1.38)	0.004
Income									
	Below $75,000+	Ref.		Ref.		Ref.		Ref.	
	Over $75,000+	0.70 (0.64–0.77)	<0.001	0.75 (0.67–0.84)	<0.001	0.76 (0.64–0.91)	0.002	0.74 (0.64–0.85)	<0.001
Rural									
	Urban	Ref.		Ref.		Ref.		Ref.	
	Rural	1.21 (1.08–1.36)	0.001	1.03 (0.89–1.19)	0.726	0.96 (0.77–1.20)	0.751	1.06 (0.87–1.30)	0.559
Primary									
	Cervix uteri	Ref.		Ref.		Ref.		Ref.	
	Endocervix	0.66 (0.60–0.73)	<0.001	0.91 (0.79–1.05)	0.209	0.89 (0.71–1.11)	0.292	0.96 (0.80–1.15)	0.668
	Exocervix	0.69 (0.51–0.93)	0.015	0.85 (0.59–1.22)	0.377	0.88 (0.51–1.51)	0.643	0.81 (0.50–1.32)	0.407
	Overlapping lesion of cervix uteri	0.82 (0.61–1.10)	0.184	0.84 (0.58–1.20)	0.329	0.70 (0.38–1.27)	0.239	0.91 (0.58–1.45)	0.699
Histology									
	Squamous	Ref.		Ref.		Ref.		Ref.	
	Adenocarcinoma	0.67 (0.61–0.74)	<0.001	1.21 (1.06–1.39)	0.006	0.93 (0.75–1.16)	0.544	1.47 (1.23–1.77)	<0.001
	Adenosquamous	0.93 (0.77–1.12)	0.440	1.30 (1.03–1.65)	0.029	1.25 (0.91–1.72)	0.162	1.27 (0.89–1.82)	0.191
Grade									
	Well differentiated	Ref.		Ref.		Ref.		Ref.	
	Moderately differentiated	2.30 (2.00–2.65)	<0.001	1.41 (1.19–1.66)	<0.001	1.57 (1.20–2.04)	<0.001	1.28 (1.02–1.60)	0.035
	Poorly differentiated	4.00 (3.47–4.60)	<0.001	1.95 (1.65–2.32)	<0.001	2.29 (1.75–2.99)	<0.001	1.64 (1.30–2.07)	<0.001
	Undifferentiated	5.31 (3.99–7.07)	<0.001	2.56 (1.81–3.61)	<0.001	2.80 (1.61–4.87)	<0.001	2.25 (1.44–3.51)	<0.001
Stage									
	Localized	Ref.		Ref.		Ref.		Ref.	
	Regional	5.30 (4.81–5.85)	<0.001	3.25 (2.88–3.66)	<0.001	4.29 (3.56–5.17)	<0.001	2.75 (2.35–3.22)	<0.001
	Distant	26.43 (22.75–30.71)	<0.001	10.00 (8.23–12.16)	<0.001	14.66 (10.67–20.15)	<0.001	8.38 (6.52–10.78)	<0.001
Tumor size									
	≤4 cm	Ref.		Ref.		Ref.		Ref.	
	>4 cm	0.33 (0.29–0.36)	<0.001	0.86 (0.75–0.97)	0.019	0.97 (0.79–1.19)	0.789	0.78 (0.66–0.93)	0.005
Lymph node									
	No	Ref.		Ref.		Ref.		Ref.	
	Yes	0.31 (0.28–0.33)	<0.001	1.08 (0.83–1.40)	0.564	1.06 (0.71–1.57)	0.783	1.03 (0.73–1.46)	0.848
Metastasis									
	No	Ref.		Ref.		Ref.		Ref.	
	Yes	3.04 (2.57–3.59)	<0.001	1.03 (0.81–1.30)	0.824	1.05 (0.72–1.52)	0.804	0.98 (0.72–1.35)	0.92
Regional nodes examined									
	No	Ref.		Ref.		Ref.		Ref.	
	Yes	0.28 (0.25–0.30)	<0.001	0.61 (0.46–0.81)	<0.001	0.59 (0.38–0.89)	0.013	0.63 (0.43–0.92)	0.017
Regional nodes positive									
	No	Ref.		Ref.		Ref.		Ref.	
	Yes	5.53 (4.99–6.13)	<0.001	1.46 (1.22–1.75)	<0.001	1.24 (0.96–1.61)	0.101	1.53 (1.19–1.97)	<0.001
Bone metastases									
	No	Ref.		Ref.		Ref.		Ref.	
	Yes	24.21 (14.05–41.71)	<0.001	2.89 (1.61–5.18)	<0.001	3.56 (1.35–9.39)	0.01	2.61 (1.27–5.35)	0.009
Brain metastases									
	No	Ref.		Ref.		Ref.			
	Yes	65.03 (8.98–471.11)	<0.001	5.07 (0.67–38.16)	0.115	1.15 (0.12–11.22)	0.905		
Liver metastases									
	No	Ref.		Ref.		Ref.		Ref.	
	Yes	37.15 (17.39–79.35)	<0.001	3.20 (1.46–7.04)	0.004	6.15 (0.80–47.02)	0.08	2.77 (1.17–6.54)	0.021
Lung metastases									
	No	Ref.		Ref.		Ref.		Ref.	
	Yes	23.55 (16.20–34.22)	<0.001	2.57 (1.70–3.88)	<0.001	5.71 (2.21–14.74)	<0.001	2.06 (1.30–3.28)	0.002

Group A*, age ≤49; Group B*, age ≥50.

### Age dependent mortality risks and associated factors in cervical cancer

A total of 3,324 deaths were recorded in the cohort, all attributed to cervical cancer, with a median follow-up time of 16 months (interquartile range: 6–32 months). The analysis of mortality patterns revealed a clear age-related trend. Restricted cubic spline (RCS) modeling demonstrated an upward trajectory in both all-cause and cancer-specific mortality with advancing age. The model controlled for confounding variables including median household income, race, geographic region, marital status, tumor site, lymph node status, histological subtype, and metastasis to bone, liver, and lung (Figures 2B,C, both *p* for non-linearity = 0.001).

**Figure 3:**
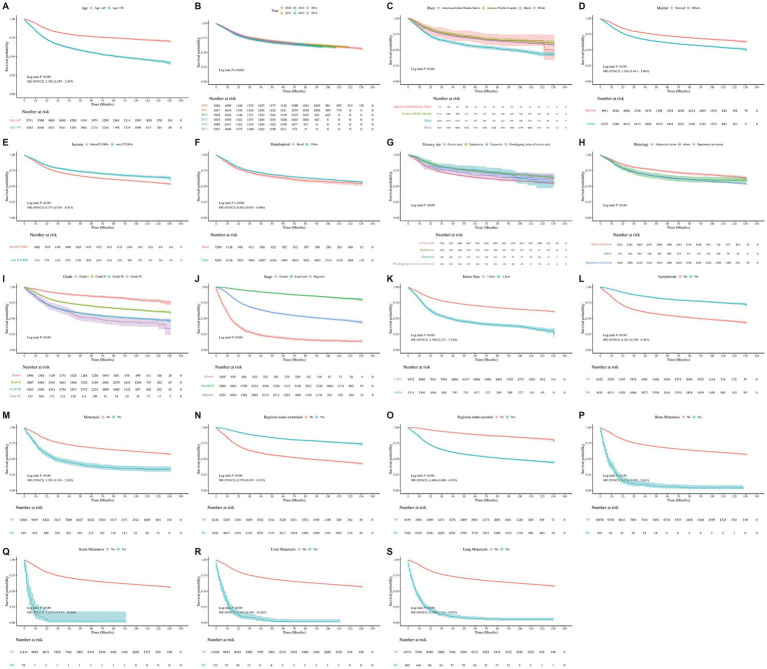
Kaplan–Meier curves representing the characteristics of each individual were plotted. **(A)** Kaplan–Meier survival curve for all-cause by age. **(B)** Kaplan–Meier survival curve for all-cause by year. **(C)** Kaplan–Meier survival curve for all-cause by race. **(D)** Kaplan–Meier survival curve for all-cause by marital. **(E)** Kaplan–Meier survival curve for all-cause by income. **(F)** Kaplan–Meier survival curve for all-cause by distributional. **(G)** Kaplan–Meier survival curve for all-cause by primary site. **(H)** Kaplan–Meier survival curve for all-cause by histology. **(I)** Kaplan–Meier survival curve for all-cause by grade. **(J)** Kaplan–Meier survival curve for all-cause by stage. **(K)** Kaplan–Meier survival curve for all-cause by tumor size. **(L)** Kaplan–Meier survival curve for all-cause by lymph node. **(M)** Kaplan–Meier survival curve for all-cause by metastasis. **(N)** Kaplan–Meier survival curve for all-cause by regional nodes examined. **(O)** Kaplan–Meier survival curve for all-cause by regional nodes positive. **(P)** Kaplan–Meier survival curve for all-cause by bone. **(Q)** Kaplan–Meier survival curve for all-cause by brain. **(R)** Kaplan–Meier survival curve for all-cause by liver. **(S)** Kaplan–Meier survival curve for all-cause by lung.

Multivariable Cox proportional hazards models further confirm age as an independent predictor of mortality. The adjusted hazard ratio (HR) for all-cause mortality per year increase in age was 1.02 (95% CI: 1.02–1.03, *p* < 0.001), and for cancer-specific mortality it was 1.01 (95% CI: 1.01–1.02, *p* < 0.001). These findings remained robust after controlling for relevant clinical and demographic covariates. Kaplan–Meier survival curves supported these trends: patients aged ≥50 years (Figure 3A) (HR = 2.342, 95% CI: 2.197–2.497, *p* < 0.001), diagnosed in earlier years (*p* < 0.001) (Figure 3B), of Black race (*p* < 0.001) (Figure 3C), unmarried (HR = 1.503, 95% CI: 1.411–1.601, *p* < 0.001) (Figure 3D), and with lower income levels (HR = 0.757, 95% CI: 0.704–0.813, *p* < 0.001) (Figure 3E) all exhibited significantly reduced survival probabilities. Similarly, rural residence (HR = 0.882, 95% CI: 0.805–0.966, *p* < 0.001) (Figure 3F), non-endocervical tumor site (*p* < 0.001) (Figure 3G), and squamous histology (*p* < 0.001) (Figure 3H) were associated with poorer outcomes. Survival differences by tumor characteristics were also apparent. Patients with poorly differentiated or undifferentiated tumors had worse survival compared to those with well-differentiated tumors (*p* < 0.001) (Figure 3I). Stage at diagnosis strongly influenced outcomes, with distant-stage disease showing the lowest survival rates (*p* < 0.001) (Figure 3J). Larger tumor size (>4 cm) paradoxically correlated with better survival (HR = 2.380, 95% CI: 2.217–2.554, *p* < 0.001) (Figure 3K), while lymph node involvement was associated with decreased survival (HR = 0.361, 95% CI: 0.336–0.387, *p* < 0.001) (Figure 3L). Patients with any distant metastasis showed markedly poorer unadjusted survival compared with those without metastasis (Figure 3M; HR = 2.41, 95% CI: 2.17–2.67, *p* < 0.001), although this association was attenuated after multivariable adjustment (adjusted HR = 0.95, 95% CI: 0.85–1.07, *p* = 0.425). Examination of regional lymph nodes was associated with better survival (Figure 3N; adjusted HR = 0.67, 95% CI: 0.56–0.81, *p* < 0.001), whereas regional nodal positivity predicted worse survival (Figure 3O; adjusted HR = 1.42, 95% CI: 1.25–1.63, *p* < 0.001). Presence of metastases to bone (HR = 8.174, 95% CI: 7.055–9.471, *p* < 0.001) (Figure 3P), brain (HR = 13.675, 95% CI: 9.913–18.866, *p* < 0.001)(Figure 3Q), liver (HR = 9.993, 95% CI: 8.459–11.805, *p* < 0.001) (Figure 3R), and lung (HR = 8.146, 95% CI: 7.314–9.072, *p* < 0.001) (Figure 3S) each significantly worsened prognosis.

Table 3 summarizes factors associated with all-cause mortality. Black race was associated with significantly increased risk compared to White individuals (HR = 1.52, 95% CI: 1.40–1.65, *p* < 0.001). Marital status emerged as another important determinant; unmarried individuals had a higher mortality risk (HR = 1.51, 95% CI: 1.41–1.60, *p* < 0.001). Socioeconomic status was protective, with income exceeding $75,000 associated with reduced mortality (HR = 0.76, 95% CI: 0.70–0.81, *p* < 0.001). Residence in rural areas corresponded to elevated risk (HR = 1.14, 95% CI: 1.04–1.24, *p* = 0.006), likely reflecting disparities in access to care.

**Table 3:** Distribution of all-cause mortality from cervical cancer and risk associated with various prognostic factors.

Variables		Deaths *N* (%)	HR (95% CI)	*p*	HR (95% CI)	*p*
Age	Continuous	4,148 (100)	1.04 (1.03–1.04)	<0.001	1.02 (1.02–1.03)	<0.001
Year						
	2010	757 (18.25)	Ref.		Ref.	
	2011	695 (16.76)	0.97 (0.87–1.07)	0.540	0.96 (0.87–1.07)	0.443
	2012	719 (17.33)	1.03 (0.93–1.14)	0.604	0.96 (0.86–1.06)	0.420
	2013	694 (16.73)	1.06 (0.95–1.17)	0.292	0.97 (0.88–1.08)	0.606
	2014	648 (15.62)	0.99 (0.89–1.10)	0.856	0.94 (0.85–1.05)	0.297
	2015	635 (15.31)	1.01 (0.90–1.12)	0.916	0.92 (0.82–1.02)	0.123
Race						
	White	3,032 (73.10)	Ref.		Ref.	
	Black	706 (17.02)	1.52 (1.40–1.65)	<0.001	1.24 (1.14–1.35)	<0.001
	American Indian/Alaska Native	41 (0.99)	0.96 (0.71–1.31)	0.797	1.07 (0.78–1.46)	0.672
	Asian or Pacific Islander	369 (8.90)	0.92 (0.83–1.03)	0.148	0.91 (0.82–1.02)	0.105
Marital						
	Married	1,527 (36.81)	Ref.		Ref.	
	Others	2,621 (63.19)	1.51 (1.41–1.60)	<0.001	1.22 (1.14–1.30)	<0.001
Income						
	Below $75,000+	3,179 (76.64)	Ref.		Ref.	
	Over $75,000+	969 (23.36)	0.76 (0.70–0.81)	<0.001	0.83 (0.77–0.90)	<0.001
Rural						
	Urban	3,616 (87.17)	Ref.		Ref.	
	Rural	532 (12.83)	1.14 (1.04–1.24)	0.006	0.98 (0.89–1.08)	0.687
Primary						
	Cervix uteri	3,416 (82.35)	Ref.		Ref.	
	Endocervix	602 (14.51)	0.69 (0.63–0.75)	<0.001	0.94 (0.85–1.03)	0.185
	Exocervix	62 (1.49)	0.73 (0.57–0.94)	0.016	0.85 (0.66–1.10)	0.222
	Overlapping lesion of cervix uteri	68 (1.64)	0.84 (0.66–1.06)	0.146	0.98 (0.77–1.24)	0.843
Histology						
	Squamous	3,161 (76.21)	Ref.		Ref.	
	Adenocarcinoma	810 (19.53)	0.71 (0.65–0.76)	<0.001	1.12 (1.02–1.22)	0.014
	Adenosquamous	177 (4.27)	0.95 (0.82–1.11)	0.515	1.20 (1.03–1.40)	0.018
Grade						
	Well differentiated	289 (6.97)	Ref.		Ref.	
	Moderately differentiated	1,675 (40.38)	2.10 (1.86–2.38)	<0.001	1.29 (1.13–1.47)	<0.001
	Poorly differentiated	2,056 (49.57)	3.30 (2.92–3.73)	<0.001	1.59 (1.40–1.81)	<0.001
	Undifferentiated	128 (3.09)	3.85 (3.13–4.74)	<0.001	1.61 (1.30–1.99)	<0.001
Stage						
	Localized	733 (17.67)	Ref.		Ref.	
	Regional	2,134 (51.45)	4.21 (3.87–4.58)	<0.001	2.70 (2.46–2.98)	<0.001
	Distant	1,281 (30.88)	13.92 (12.69–15.26)	<0.001	6.36 (5.67–7.14)	<0.001
Tumor size						
	≤4 cm	1,028 (24.78)	Ref.		Ref.	
	>4 cm	3,120 (75.22)	0.42 (0.39–0.45)	<0.001	0.94 (0.87–1.01)	0.083
Lymph node						
	No	3,072 (74.06)	Ref.		Ref.	
	Yes	1,076 (25.94)	0.36 (0.34–0.39)	<0.001	1.04 (0.88–1.23)	0.655
Metastasis						
	No	3,760 (90.65)	Ref.		Ref.	
	Yes	388 (9.35)	2.41 (2.17–2.67)	<0.001	0.95 (0.85–1.07)	0.425
Regional nodes examined						
	No	3,125 (75.34)	Ref.		Ref.	
	Yes	1,023 (24.66)	0.33 (0.31–0.35)	<0.001	0.67 (0.56–0.81)	<0.001
Regional nodes positive						
	No	530 (12.78)	Ref.		Ref.	
	Yes	3,618 (87.22)	4.47 (4.08–4.89)	<0.001	1.42 (1.25–1.63)	<0.001
Bone metastases						
	No	3,957 (95.40)	Ref.		Ref.	
	Yes	191 (4.60)	8.45 (7.29–9.79)	<0.001	1.69 (1.44–1.98)	<0.001
Brain metastases						
	No	4,110 (99.08)	Ref.		Ref.	
	Yes	38 (0.92)	14.58 (10.57–20.11)	<0.001	2.84 (2.04–3.95)	<0.001
Liver metastases						
	No	4,000 (96.43)	Ref.		Ref.	
	Yes	148 (3.57)	10.42 (8.82–12.31)	<0.001	1.92 (1.60–2.30)	<0.001
Lung metastases						
	No	3,768 (90.84)	Ref.		Ref.	
	Yes	380 (9.16)	8.39 (7.53–9.34)	<0.001	1.60 (1.41–1.82)	<0.001

Histopathological variables also influenced mortality. Patients with endocervical tumor location had a lower risk (HR = 0.69, 95% CI: 0.63–0.75, *p* < 0.001), as did those with adenocarcinoma histology (HR = 0.71, 95% CI: 0.65–0.76, *p* < 0.001). In contrast, poorly differentiated tumors (HR = 3.30, 95% CI: 2.92–3.73, *p* < 0.001) and distant-stage disease (HR = 13.92, 95% CI: 12.69–15.26, *p* < 0.001) conferred markedly increased mortality risk. Interestingly, tumor size >4 cm (HR = 0.42, 95% CI: 0.39–0.45, *p* < 0.001) and lymph node involvement (HR = 0.36, 95% CI: 0.34–0.39, *p* < 0.001) were inversely associated with all-cause mortality, possibly reflecting treatment selection or residual confounding. However, bone metastasis significantly elevated mortality (HR = 8.45, 95% CI: 7.29–9.79, *p* < 0.001), highlighting its aggressive clinical implication.

Table 4 outlines factors associated with cancer-specific mortality. The pattern largely mirrored that of all-cause mortality. Black individuals exhibited increased risk (HR = 1.47, 95% CI: 1.34–1.61, *p* < 0.001), as did those who were unmarried (HR = 1.44, 95% CI: 1.34–1.54, *p* < 0.001). High-income status continued to show a protective effect (HR = 0.76, 95% CI: 0.70–0.82, *p* < 0.001), and rural residence remained a risk factor (HR = 1.11, 95% CI: 1.01–1.23, *p* = 0.042).

**Table 4:** Distribution of cervical cancer-specific mortality and hazard risk associated with various prognostic factors.

Variables		Deaths *N* (%)	HR (95% CI)	*p*-value	HR (95% CI)	*p*-value
Age	Continuous	3,324 (100)	1.03 (1.03–1.03)	<0.001	1.01 (1.01–1.02)	<0.001
Year						
	2010	579 (17.42)	Ref.		Ref.	
	2011	528 (15.88)	0.94 (0.83–1.06)	0.298	0.94 (0.83–1.06)	0.296
	2012	574 (17.27)	1.03 (0.91–1.15)	0.662	0.95 (0.84–1.06)	0.344
	2013	578 (17.39)	1.08 (0.96–1.22)	0.178	0.98 (0.87–1.10)	0.771
	2014	548 (16.49)	1.01 (0.90–1.14)	0.849	0.95 (0.84–1.07)	0.395
	2015	517 (15.55)	0.97 (0.86–1.10)	0.649	0.87 (0.78–0.99)	0.029
Race						
	White	2,420 (72.80)	Ref.		Ref.	
	Black	551 (16.58)	1.47 (1.34–1.61)	<0.001	1.20 (1.09–1.32)	<0.001
	American Indian/Alaska Native	36 (1.08)	1.06 (0.76–1.48)	0.717	1.13 (0.81–1.57)	0.469
	Asian or Pacific Islander	317 (9.54)	0.99 (0.88–1.11)	0.878	1.01 (0.89–1.14)	0.898
Marital						
	Married	1,255 (37.76)	Ref.		Ref.	
	Others	2,069 (62.24)	1.44 (1.34–1.54)	<0.001	1.19 (1.11–1.28)	<0.001
Income						
	Below $75,000+	2,550 (76.71)	Ref.		Ref.	
	Over $75,000+	774 (23.29)	0.76 (0.70–0.82)	<0.001	0.84 (0.77–0.91)	<0.001
Rural						
	Urban	2,905 (87.39)	Ref.		Ref.	
	Rural	419 (12.61)	1.11 (1.01–1.23)	0.042	0.95 (0.86–1.06)	0.371
Primary						
	Cervix uteri	2,755 (82.88)	Ref.		Ref.	
	Endocervix	467 (14.05)	0.67 (0.61–0.74)	<0.001	0.92 (0.82–1.02)	0.114
	Exocervix	46 (1.38)	0.67 (0.50–0.90)	0.008	0.82 (0.62–1.10)	0.195
	Overlapping lesion of cervix uteri	56 (1.68)	0.86 (0.66–1.12)	0.269	1.04 (0.80–1.36)	0.760
Histology						
	Squamous	2,508 (75.45)	Ref.		Ref.	
	Adenocarcinoma	657 (19.77)	0.73 (0.67–0.80)	<0.001	1.17 (1.06–1.29)	0.001
	Adenosquamous	159 (4.78)	1.08 (0.92–1.26)	0.364	1.29 (1.09–1.52)	0.002
Grade						
	Well differentiated	206 (6.20)	Ref.		Ref.	
	Moderately differentiated	1,302 (39.17)	2.27 (1.96–2.62)	<0.001	1.29 (1.11–1.50)	0.001
	Poorly differentiated	1,708 (51.38)	3.77 (3.26–4.35)	<0.001	1.63 (1.40–1.90)	<0.001
	Undifferentiated	108 (3.25)	4.44 (3.51–5.60)	<0.001	1.69 (1.33–2.14)	<0.001
Stage						
	Localized	419 (12.61)	Ref.		Ref.	
	Regional	1,712 (51.50)	5.76 (5.18–6.42)	<0.001	3.89 (3.45–4.38)	<0.001
	Distant	1,193 (35.89)	21.34 (19.07–23.88)	<0.001	10.28 (8.96–11.80)	<0.001
Tumor size						
	≤4 cm	857 (25.78)	Ref.		Ref.	
	>4 cm	2,467 (74.22)	0.41 (0.38–0.44)	<0.001	0.95 (0.87–1.03)	0.190
Lymph node						
	No	2,487 (74.82)	Ref.		Ref.	
	Yes	837 (25.18)	0.35 (0.33–0.38)	<0.001	1.03 (0.86–1.24)	0.753
Metastasis						
	No	2,966 (89.23)	Ref.		Ref.	
	Yes	358 (10.77)	2.76 (2.47–3.08)	<0.001	0.94 (0.83–1.06)	0.328
Regional nodes examined						
	No	2,533 (76.20)	Ref.		Ref.	
	Yes	791 (23.80)	0.32 (0.30–0.35)	<0.001	0.67 (0.55–0.82)	<0.001
Regional nodes positive						
	No	356 (10.71)	Ref.		Ref.	
	Yes	2,968 (89.29)	5.32 (4.76–5.93)	<0.001	1.45 (1.25–1.69)	<0.001
Bone						
	No	3,140 (94.46)	Ref.		Ref.	
	Yes	184 (5.54)	9.55 (8.22–11.11)	<0.001	1.68 (1.43–1.98)	<0.001
Brain						
	No	3,286 (98.86)	Ref.		Ref.	
	Yes	38 (1.14)	16.93 (12.26–23.37)	<0.001	2.74 (1.97–3.82)	<0.001
Liver						
	No	3,180 (95.67)	Ref.		Ref.	
	Yes	144 (4.33)	11.75 (9.92–13.93)	<0.001	1.93 (1.61–2.33)	<0.001
Lung						
	No	2,962 (89.11)	Ref.		Ref.	
	Yes	362 (10.89)	9.51 (8.51–10.63)	<0.001	1.66 (1.46–1.90)	<0.001

Similar to the findings for overall mortality, endocervical tumor location (HR = 0.67, 95% CI: 0.61–0.74, *p* < 0.001) and adenocarcinoma histology (HR = 0.73, 95% CI: 0.67–0.80, *p* < 0.001) were associated with lower cancer-specific mortality. Poorly differentiated tumors (HR = 3.77, 95% CI: 3.26–4.35, *p* < 0.001) and distant-stage disease (HR = 21.34, 95% CI: 19.07–23.88, *p* < 0.001) again emerged as dominant predictors of poor outcome. Bone metastasis was strongly associated with increased cancer-specific mortality (HR = 8.45, 95% CI: 7.29–9.79, *p* < 0.001), consistent with its clinical significance.

### Hazard model reveals key cervical cancer outcomes

The hazard model for proportional sub-distribution of cervical cancer outcomes revealed several significant factors associated with cancer-specific mortality (Table 5). Race played a notable role, with Black women exhibiting a higher risk of mortality compared to White women (adjusted sHR = 1.199, 95% CI: 1.086–1.323, *p* = 0.0003). Socioeconomic status was also influential, as individuals with annual household income above $75,000 demonstrated a lower risk of mortality (adjusted sHR = 0.842, 95% CI: 0.772–0.919, *p* = 0.0001) relative to those with lower income levels.

**Table 5:** Hazard model for proportional sub-distribution of cervical cancer.

Variables		Univariable sHR (95% CI)	*p*-value	Multivariable sHR (95% CI)	*p*-value
Age	Continuous				
Year					
	2010	Ref.		Ref.	
	2011	0.939 (0.835–1.057)	0.2989	0.92 (0.812–1.043)	0.1929
	2012	1.026 (0.914–1.152)	0.6603	0.94 (0.831–1.064)	0.3298
	2013	1.083 (0.964–1.216)	0.1795	0.99 (0.874–1.121)	0.8701
	2014	1.012 (0.899–1.138)	0.8485	0.945 (0.834–1.071)	0.3754
	2015	0.973 (0.863–1.096)	0.6499	0.868 (0.766–0.985)	0.0277
Race					
	White	Ref.		Ref.	
	Black	1.467 (1.338–1.609)	<0.0001	1.199 (1.086–1.323)	0.0003
	American Indian/Alaska Native	1.063 (0.765–1.476)	0.7176	1.096 (0.802–1.497)	0.5654
	Asian or Pacific Islander	0.991 (0.883–1.112)	0.8773	1.005 (0.888–1.137)	0.9373
Marital					
	Married	Ref.		Ref.	
	Others	1.433 (1.337–1.537)	<0.0001	1.165 (1.081–1.256)	<0.0001
Income					
	below $75,000+	Ref.		Ref.	
	over $75,000+	0.756 (0.698–0.819)	<0.0001	0.842 (0.772–0.919)	0.0001
Rural					
	Urban	Ref.		Ref.	
	Rural	1.111 (1.003–1.232)	0.0441	0.958 (0.856–1.073)	0.4584
Primary					
	Cervix uteri	Ref.		Ref.	
	Endocervix	0.673 (0.611–0.7)	<0.0001	0.928 (0.83–1.038)	0.1932
	Exocervix	0.675 (0.507–0.9)	0.0073	0.821 (0.606–1.112)	0.2027
	Overlapping lesion of cervix uteri	0.862 (0.661–1.124)	0.2714	1.044 (0.804–1.357)	0.7453
Histology					
	Squamous	Ref.		Ref.	
	Adenocarcinoma	0.731 (0.671–0.796)	<0.0001	1.185 (1.07–1.313)	0.0011
	Adenosquamous	1.077 (0.918–1.263)	0.3632	1.273 (1.071–1.512)	0.0061
Grade					
	Well differentiated	Ref.		Ref.	
	Moderately differentiated	2.261 (1.954–2.615)	<0.0001	1.333 (1.148–1.548)	0.0002
	Poorly differentiated	3.75 (3.249–4.328)	<0.0001	1.667 (1.432–1.941)	<0.0001
	Undifferentiated	4.415 (3.512–5.55)	<0.0001	1.749 (1.363–2.244)	<0.0001
Stage					
	Localized	Ref.		Ref.	
	Regional	5.745 (5.171–6.382)	<0.0001	3.971 (3.517–4.483)	<0.0001
	Distant	20.998 (18.758–23.507)	<0.0001	10.635 (9.207–12.285)	<0.0001
Tumor size					
	≤4 cm	Ref.		Ref.	
	>4 cm	0.409 (0.379–0.442)	<0.0001	0.956 (0.874–1.045)	0.3209
Lymph node					
	No	Ref.		Ref.	
	Yes	0.354 (0.328–0.382)	<0.0001	1.023 (0.822–1.271)	0.8413
Metastasis					
	No	Ref.		Ref.	
	Yes	2.743 (2.452–3.068)	<0.0001	0.95 (0.828–1.089)	0.4609
Regional nodes examined					
	No	Ref.		Ref.	
	Yes	0.321 (0.297–0.348)	<0.0001	0.67 (0.532–0.845)	0.0007
Regional nodes positive					
	No	Ref.		Ref.	
	Yes	5.294 (4.752–5.897)	<0.0001	1.427 (1.228–1.659)	<0.0001
Bone					
	No	Ref.		Ref.	
	Yes	9.243 (7.857–10.874)	<0.0001	1.711 (1.404–2.084)	<0.0001
Brain					
	No	Ref.		Ref.	
	Yes	15.846 (10.59–23.708)	<0.0001	2.774 (1.78–4.323)	<0.0001
Liver					
	No	Ref.		Ref.	
	Yes	11.286 (9.483–13.433)	<0.0001	1.869 (1.512–2.31)	<0.0001
Lung					
	No	Ref.		Ref.	
	Yes	9.235 (8.148–10.468)	<0.0001	1.707 (1.468–1.984)	<0.0001

Marital status emerged as another significant predictor, with unmarried individuals facing a higher risk of death (adjusted sHR = 1.165, 95% CI: 1.081–1.256, *p* < 0.0001) compared to their married counterparts. Histological subtypes were associated with differential risks; patients with adenocarcinoma (adjusted sHR = 1.185, 95% CI: 1.070–1.313, *p* = 0.0011) and adenosquamous carcinoma (adjusted sHR = 1.273, 95% CI: 1.071–1.512, *p* = 0.0061) exhibited significantly higher mortality risks than those with squamous cell carcinoma.

The cancer stage was the strongest predictor of adverse outcomes. Compared to patients diagnosed at a localized stage, those with regional stage disease had a nearly fourfold increased risk (adjusted sHR = 3.971, 95% CI: 3.517–4.483, *p* < 0.0001), while distant stage disease conferred an over tenfold increased risk (adjusted sHR = 10.635, 95% CI: 9.207–12.285, *p* < 0.0001). Tumor grade was similarly predictive of outcomes. Poorly differentiated tumors were associated with elevated risk (adjusted sHR = 1.667, 95% CI: 1.432–1.941, *p* < 0.0001), and undifferentiated tumors showed an even greater risk (adjusted sHR = 1.749, 95% CI: 1.363–2.244, *p* < 0.0001), relative to well-differentiated tumors.

## Discussion

This study provides a comprehensive analysis of age-related patterns in cervical cancer incidence and mortality using SEER database data. Our findings reveal significant disparities influenced by demographic, socioeconomic, and clinical factors that are essential for guiding public health strategies to improve CC outcomes across diverse populations.

Our analysis identified a distinct U-shaped relationship between age and CC incidence, with peaks occurring in both younger and older age groups. This pattern highlights two key intervention windows, early adulthood and post-menopausal age. The increased incidence among younger women likely reflects early initiation of sexual activity, higher rates of HPV infection, and potentially lower uptake of vaccination or screening in specific populations, consistent with the previous studies (31–33). In contrast, the higher incidence among older women may be due to the cumulative effects of persistent HPV infection and limitations of current screening programs, which often become less effective as women age (34, 35). These findings suggest that guidelines for CC prevention and screening may warrant reassessment to better address the continued risk of older women.

Mortality rates demonstrated significant variation with age, with older women exhibiting higher all-cause and cancer-specific mortality. This trend aligns with existing literature, which has shown that older women are more often diagnosed at advanced stages and tend to have additional comorbidities, both of which complicate treatment and adversely affect survival rates (36). The elevated mortality risk in this demographic underscores the need for targeted screening and treatment strategies that consider the unique challenges encountered by older women (37).

Our research further corroborates the persistence of substantial racial and socioeconomic disparities in CC outcomes. Black women, in particular, continue to experience higher incidence and mortality compared to White women, and this gap is widening in older age groups. These inequalities arise from multifactorial sources, including unequal access to healthcare, socioeconomic disadvantage, and, to a lesser extent, possible biological differences in tumor characteristics (38). Importantly, Black women face greater barriers to regular screening and prompt treatment, resulting in a higher likelihood of late-stage diagnoses and poorer outcomes (39). These findings highlight the urgent imperative for policies and interventions specifically aimed at reducing healthcare disparities.

Income and marital status emerged as significant determinants of CC outcomes. Higher income was associated with lower mortality, likely reflecting better healthcare access and healthier lifestyles (40). Unmarried women had higher mortality rates, possibly linked to reduced social support and lower healthcare engagement (41). These findings emphasize the importance of addressing social determinants of health.

Analysis of clinical and pathological characteristics revealed that tumor grade and stage are among the most powerful predictors of risk and mortality associated with CC. Poorly differentiated tumors and those diagnosed at advanced stages are strongly associated with higher CC odds and increased mortality (42). The observed inverse relationship between tumor size and cervical cancer risk may reflect tumor biology complexities and detection biases (43, 44). Notably, lymph node involvement was not a significant independent predictor for CC risk in the multivariable model. This finding is in line with some recent studies suggesting that other factors, such as molecular tumor features and patient demographics, may play a more defining role in outcomes (45). However, metastasis to distant organs such as bone, brain, liver, and lung was strongly associated with elevated mortality risk, emphasizing the need for comprehensive staging and tailored treatment plans (46, 47).

The observed U-shaped incidence curve suggests that screening strategies should address the specific needs of both younger and older women. For younger women, increasing HPV vaccination coverage and promoting regular screening remain essential (48, 49). For older women, extending the age range for routine screening and ensuring that programs remain accessible may be especially essential (50). The persistent disparities found in our study highlight the call for targeted interventions, including community-based outreach, culturally tailored education, and policy changes to reduce barriers to healthcare access (51). Integrating social support services into cancer care can help address higher mortality risks among unmarried and lower-income women (52).

### Strengths and limitations

This study’s strengths lie in its large, population-based cohort and the comprehensive nature of the SEER database, which provides detailed demographic, clinical, and survival data. The use of advanced statistical techniques, such as restricted cubic splines and competing risk models, allowed for a nuanced analysis of age-incidence and age-mortality relationships. However, several limitations must be acknowledged. The retrospective nature of the study may introduce selection and information biases. Although the SEER database is extensive, it covers only about 28% of the U.S. population, which may limit the generalizability of the findings to other regions or populations not represented in the database. A noticeable limitation is the absence of HPV vaccination status, which is not captured in SEER. HPV vaccination has been shown to reduce invasive cervical cancer incidence by approximately 50–90%, particularly when administered before age 17, and real-world declines in cervical cancer have already been observed in vaccinated cohorts. Its omission restricts our ability to adjust for one of the most significant protective and confounding factors in cervical cancer epidemiology. The impact is particularly relevant for younger cohorts, where vaccine uptake varies by age, race/ethnicity, and socioeconomic status. Such heterogeneity may partly explain the observed disparities in incidence and survival in our analyses. Therefore, variations in vaccination uptake across age, race, and socioeconomic groups could influence the observed associations in our analysis. While this limitation is inherent to SEER-based studies, it is essential to interpret our findings with this potential bias in mind. Future studies integrating immunization records with cancer registries would offer a more comprehensive understanding of these interactions.

## Conclusion

This study reveals important age-related patterns in cervical cancer incidence and mortality, highlighting significant disparities based on race, socioeconomic status, and clinical factors. The findings emphasize the need for tailored screening and prevention strategies for different age groups and high-risk populations. Focusing on early detection, equitable care access, and targeted interventions can improve outcomes and reduce the burden of this preventable disease. Continued research and public health efforts are crucial for advancing the fight against cervical cancer.

## Data Availability

Publicly available datasets were analyzed in this study. This data can be found here: the datasets used during the current study are available at https://seer.cancer.gov.

## Footnotes

^1^ www.zstats.net
